# CXCL10/IP-10 Neutralization Can Ameliorate Lipopolysaccharide-Induced Acute Respiratory Distress Syndrome in Rats

**DOI:** 10.1371/journal.pone.0169100

**Published:** 2017-01-03

**Authors:** Shan Lang, Libing Li, Xuning Wang, Junping Sun, Xinying Xue, Yongjiu Xiao, Mingyue Zhang, Ting Ao, Jianxin Wang

**Affiliations:** 1 Department of Respiratory Diseases, Chinese PLA General Hospital, Beijing, China; 2 Department of Cardiovascular Surgery, Chinese PLA General Hospital, Beijing, China; 3 Department of Surgical Oncology, Chinese PLA General Hospital, Beijing, China; Centre National de la Recherche Scientifique, FRANCE

## Abstract

The role of C-X-C motif chemokine 10 (CXCL10), a pro-inflammatory factor, in the development of acute respiratory distress syndrome (ARDS) remains unclear. In this study, we explored the role of CXCL10 and the effect of CXCL10 neutralization in lipopolysaccharide (LPS)-induced ARDS in rats. The expression of CXCL10 and its receptor chemokine receptor 3(CXCR3) increased after LPS induction. Moreover, neutralization of CXCL10 ameliorated the severity of ARDS by reducing pulmonary edema, inhibiting the release of inflammatory mediators (IFN-γ, IL-6 and ICAM-1) and limiting inflammatory cells (neutrophils, macrophages, CD8+ T cells) influx into the lung, with a reduction in CXCR3 expression in neutrophils and macrophages. Therefore, CXCL10 could be a potential therapeutic target in LPS-induced ARDS.

## Introduction

Acute respiratory distress syndrome (ARDS) is a fatal disease triggered by multiple conditions including severe sepsis and pneumonia [1, 2]. Despite significant advances in therapeutic strategies including mechanical ventilation and pharmacotherapy, the mortality rate for ARDS remains high [3–5]. Previous studies have shown that ARDS is associated with a high production of pro-inflammatory cytokines and chemokines, such as TNF-α, IL-1β, and IL-6 [6, 7]. In addition, the excessive activation and recruitment of neutrophils into inflamed lungs exacerbates the pathogenesis of ARDS and may indicate a poor clinical outcome [8, 9]. Neutrophils migration to the lung is mediated by various factors, among which chemokines and cell adhesion molecules are considered the most important [10]. The C-X-C motif chemokine10(CXCL10), also known as interferon-γ inducible protein 10 (IP-10), is a chemokine that modulates innate and adaptive immune responses by recruiting inflammatory cells (i.e., neutrophils, T lymphocytes and NK cells) to the sites of inflammation [11]. By binding to its receptor CXCR3, CXCL10 can induce chemotaxis, apoptosis, cell growth and angiostasis [12]. Previous studies have shown that chemokines and their receptors play an essential role in various infectious diseases [13]. The three CXCR3 ligands (CXCL9, CXCL10 and CXCL11) are known to be differentially elevated under many conditions, such as interstitial cystitis, ulcerative colitis, and myositis; moreover, blocking CXCL10 may ameliorate the severity of these diseases [14–16]. Patients infected with ARDS also show unusually high levels of CXCL10 and uncontrolled inflammation is associated with the development of ARDS [17–19]. However, the mechanism of CXCL10 on the development of ARDS remains unclear.

In the present study, we established an ARDS experimental model through intratracheal instillation of lipopolysaccharide (LPS) derived from components of the Gram-negative bacteria wall. This model has been widely used [20, 21]. It has documented that LPS can trigger ARDS by increasing inflammatory cytokines production in lung tissues. Based on microarrays, it has been shown that the CXCL10 gene is upregulated after LPS stimulation in acute lung injury [22]. Therefore, we used this model to explore whether CXCL10 contributes to the pathophysiology of ARDS induced by LPS. To further elucidate the effect of CXCL10, anti-CXCL10 antibody was used to neutralize the chemokine CXCL10 in our ARDS model. To this end, we first assessed the blood oxygenation and pulmonary histopathology in rats after LPS induction to ensure that the ARDS model has been established. The expression of CXCL10 and CXCR3 in our model was then measured. Finally, anti-CXCL10 antibody was administered to determine whether CXCL10 neutralization can ameliorate LPS-induced ARDS and to explore the molecular mechanisms relating ARDS.

## Materials and Methods

### LPS-induced ARDS model in rats

Male Wistar rats weighing 180–220 g were purchased from the Academy of Military Medical Sciences Laboratory Animal Center (Beijing, China). The ARDS model was established as described previously [20]. After the rats were anesthetized with 3% sodium pentobarbital, LPS (Escherichia coli O111:B4; Sigma, St. Louis, MO, USA) dissolved in saline was instilled slowly into the tracheas at a dose of 2 mg/kg to induce ARDS. Rats in the control group were administered an equal volume of saline. The rats were then placed upright to ensure that LPS or saline distributed equally in bilateral lung tissues. At 2, 6 and 12 h after exposure to LPS or saline, blood was collected from the abdominal aorta. For neutralizing CXCL10, rats were administered anti-CXCL10 antibody (R&D System, Minneapolis, MN) or isotype-matched anti-IgG1 antibody (R&D Systems, Minneapolis, MN, USA) with an intraperitoneal injection (50 μg in 100 μL saline per rat) 30 min prior to LPS administration and 1 h after LPS administration.

All protocols were conducted in accordance with the Guidelines for Animal Experimentation. The rats had free access to water and food and adapted to the experimental environment for 2 days before carrying out the experiments. The rats were maintained in a room with 12 h dark/light cycles and 40–60% humidity. During the study, the rats were monitored every 1 h to evaluate their physical condition. When rats showed signs of agonal breathing or no response to touch, they were humanely euthanized with an overdose of sodium pentobarbital. Sodium pentobarbital anesthesia was essential before performing surgery, and all efforts were made to minimize suffering. This study was approved by the Ethical Committee on Animal Research at Chinese PLA General Hospital.

### Pulmonary histopathology

The right upper lobe of the lung was excised and fixed with 10% neutral buffered formalin for 48 h. After fixation, the tissues were dehydrated and embedded in paraffin. Samples were then cut into 5μm sections, stained with hematoxylin and eosin (H&E) and examined using light microscopy. A semi-quantitative scoring system was used to assess histopathological changes in lung tissues as described previously [23], which included five different variables, including alveolar and interstitial inflammation, pulmonary hemorrhage, edema, atelectasis and formation of hyaline membranes. Each variable was scored from 0 to 4: no injury scored 0; 1 was injury to less than 25% of the field; 2 was injury to no more than 50% of the field but beyond 25%; 3 was injury to more than 50% but less than 75% of the field; and 4 was diffuse injury. The scores of each variable were evaluated by two experienced pathologists who were blinded to the experimental procedures. Scores were added for the five variables in the same sample, with a maximum possible score of 20.

### Arterial blood gas

Blood samples from the abdominal aorta were obtained after 2, 6 and 12 h of LPS or saline intratracheal instillation. PaO_2_ was measured using the AVL OMNI Blood Gas Analyzer (Switzerland) as an indicator for assessing respiratory failure.

### BALF collection

Bronchoalveolar lavage fluid (BALF) samples were obtained as described previously [24]. Briefly, the left lung was flushed three times with 2mL of saline through a tracheal cannula. The total recovery rate was more than 90%. The BALF samples were immediately centrifuged at 3000 rpm for 10 min at 4°C. The supernatant fluids were stored at −80°C prior to the cytokine assay.

### ELISA

Blood samples (1mL) from the abdominal aorta were centrifuged at 3000 rpm for 10 min. Serum and BALF concentrations of CXCL9 (MyBioSource), CXCL10 (MyBioSource) and CXCL11(LifeSpan BioSciences Inc) in rats were measured with enzyme-linked immunosorbent assay (ELISA) kits according to the manufacturer’s instructions. Briefly, 100μL samples or standards were added to each well and incubated for 2h (CXCL9) or 90 min (CXCL10 and CXCL11) at 37°C. Biotinylated Detection Antibody (100 μL) were added and incubated for 1 h at 37°C. Horseradish peroxidase (HRP) conjugate (100 μL) was added and incubated for 30 min at 37°C, and 90μL of Tetramethylbenzidine (TMB) substrate solution was added and incubated for 15 min at 37°C. Finally, 50 μL of stop solution was added and the values were read immediately at 450 nm.

### Flow cytometry

BALF were harvested for cell analysis using a FACSCalibur flow cytometer (BD Biosciences, San Jose, CA, USA). Cells were stained with antibodies, as shown in **Table 1**. Cell staining was performed according to the protocols. Isotype controls were included to ensure antibody specificity. Cells were incubated with 5μL of antibody for 10 min in the dark at 4°C prior to permeabilization. After staining, the cells were washed three times and analyzed using CellQuest software (BD Biosciences). We first calculated the percentages of granulocytes, macrophages and T lymphocytes in total leukocytes, after which the two subsets (CD3+CD4+ and CD3+CD8+ T cells) were calculated from the total T lymphocytes. Finally, the proportion of CXCR3 positivity in inflammatory cells was investigated.

**Table 1:** Monoclonal antibodies used for the analysis of inflammatory cells.

Antibody	Fluorochrome	Supplier	Clone	Catalogue number	Dilution
CD4	PITC	Miltenyi Biotec	REA482	130-107-667	1 in 100
CD8	PE	Miltenyi Biotec	REA222	130-103-322	1 in 100
CD3	PerCP-Vio700	Miltenyi Biotec	REA223	130-103-128	1 in 100
CD11b/c	PE-Vio770	Miltenyi Biotec	REA325	130-105-318	1 in 100
CD68	PE	Miltenyi Biotec	REA237	130-103-363	1 in 100
REA Control	PE	Miltenyi Biotec	REA293	130-104-628	1 in 100
Anti-Granulocytes	FITC	Miltenyi Biotec	REA535	130-108-119	1 in 100
REA Control	FITC	Miltenyi Biotec	REA293	130-104-626	1 in 100
CXCR3	APC	R&D Systems	868013	FAB8109A	1 in 100
Isotype Control	APC	R&D Systems	133303	IC0041A	1 in 100

### Lung wet/dry weight ratio

After 12 h, rats were sacrificed and their right middle lobe lungs were immediately weighed to obtain the “wet” weight. Subsequently, the tissues were dehydrated to determine the “dry” weight in an oven at 80°C for 72 h. Lung wet/dry weight ratio was calculated and lung edema scores were evaluated [23] to assess the severity of pulmonary edema.

### Western blotting

The protein expression of CXCL10 and CXCR3 were evaluated by Western blotting. Briefly, total proteins were extracted from lung tissues using RIPA Buffer (SinoGene) according to the manufacturer’s instructions. Protein concentrations were determined using the Bradford Protein Assay Reagent (SinoGene). After blocking with Fast Protein-free Block Buffer (SinoGene), the membranes were incubated with primary antibody CXCL10 or CXCR3 (Abcam, 1:1000) at room temperature for 3 h. Subsequently, secondary antibody (horseradish peroxidase-conjugated goat anti-mouse immunoglobulin, 1:3000) was incubated at 37°C for 1 h. Protein bands were exposed with enhanced ECL kits and the relative protein levels of CXCL10 and CXCR3 were normalized to β-actin.

### Quantitative PCR

The right lower lobe of the lung was obtained and frozen in liquid nitrogen immediately prior to analysis of mRNA expression levels. Trizol reagent (Invitrogen, Carlsbad, CA, USA) was used to extract total RNA from lung tissues according to the manufacturer’s instructions. Total RNA concentration was determined using NanoDrop2000 (Thermo Scientific, USA). A two-step reaction process, that is, reverse transcription (RT) and polymerase chain reaction (PCR), was performed to quantify mRNA levels. RT was performed using the GeneAmp® PCR System 9700 (Applied Biosystems, USA) and PCR with LightCycler® 480 Ⅱ Real-time PCR Instrument (Roche, Swiss). The reaction mixture included a cDNA template, QuantiFast® SYBR® Green PCR Master Mix (Qiagen, Germany), gene-specific primers and nuclease-free water. The primer sequences were synthesized by Generay Biotech (Generay, PRC), which were based on mRNA sequences obtained from the National Center for Biotechnology Information (NCBI) database (**Table 2**). The relative expression levels of mRNA were calculated using the 2^−∆∆Ct^ method, with β -actin as a reference gene.

**Table 2:** Primers used in qPCR.

Gene	NCBI accession	Primer sequences	Product length(bp)	Ta(°C)
CXCL10	NM_139089	F:TTCCGTAAGCTATGTGCAGGTA	112	60
		R:TCAGGTGAACTCAGAACTGATG		
CXCR3	NM_053415	F: AGCACATCTCCCTACGATTA	102	60
		R: TGGCAGGAAGGTTCTGTC		
IL-6	NM_012589	F: CACAGAAGGAGTGGCTAAG	105	60
		R: TAGCACACTAGGTTTGCCG		
IL-10	NM_012854	F: AAGCTGAAGACCCTCTGGATA	126	60
		R: CTTGTAGACACCTTTGTCTTGG		
INF-γ	NM_138880	F: CTGTTACTGCCAAGGCAC	122	60
		R: TTTGCCAGTTCCTCCAGAT		
ICAM-1	NM_012967	F: GAGGATCACAAACGACGC	111	60
		R: GTCCAGGTGAGGACCATA		
β-actin	NM_031144.2	F: CCACCATGTACCCAGGCATT	189	60
		R: CGGACTCATCGTACTCCTGC		

### Statistical analysis

SPSS 19.0 was used for the statistical analyses (SPSS Inc., Chicago, USA). All continuous variables were checked for normality using the Kolmogorov-Smirnov test and homogeneity using Bartlett’s test. When data showed normal distribution and equal variance, comparisons between the two groups were performed using the Student’s t test and comparisons among multiple groups was performed using one-way analysis of variance(ANOVA). When the data showed unequal variance, the Kruskal–Wallis test, a nonparametric test was performed. Either the χ^2^ test or Fisher’s exact test was used for the discrete variables. A value of p<0.05 was considered statistically significant.

## Results

### Establishment of the ARDS model by LPS induction

**Fig 1:**
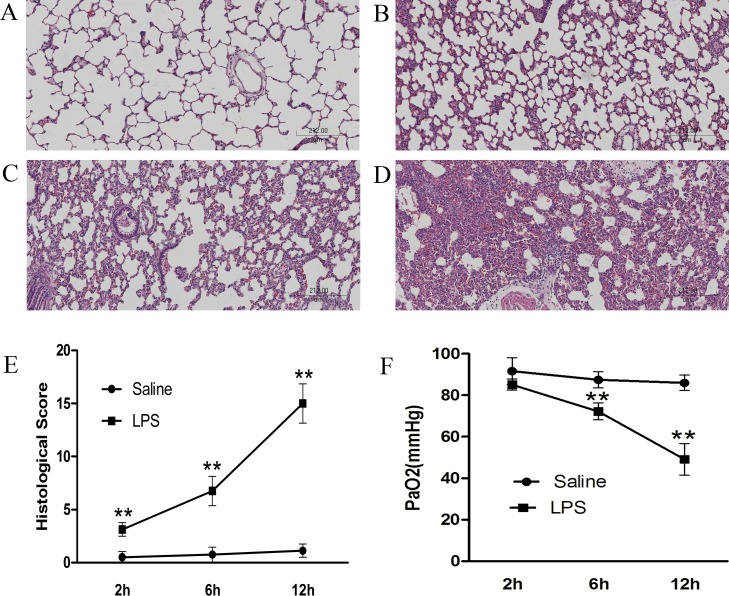
Changes in lung histopathology and PaO_2_ after intratracheal instillation of LPS or saline in rats. After 2, 6 or 12 h interventions, the right upper lobe of the lung was fixed and tissue sections were stained with H&E. Representative histological changes in lung tissues obtained from (A) the saline group and LPS groups (B: LPS 2 h, C: LPS 6 h, D: LPS 12 h). (E) Semi-quantification of histological changes and (F) PaO_2_ changes demonstrated that lung injury worsened with increased time. The results are presented as mean±SD (n = 8 each group). **p<0.01 compared with control rats at the same time point. LPS: lipopolysaccharide; H&E: hematoxylin and eosin; SD: standard deviation.

To assess lung injury, we observed pathological changes in lung tissues and measured PaO_2_ in arterial blood. As shown in **Fig 1**, there was a significant difference between the saline group and LPS groups. Lung tissues obtained from the saline group appeared normal with no significant signs of inflammatory cells infiltration or interstitial edema (**Fig 1A**). In contrast, lung tissues in LPS groups showed extensive lung injury that worsened over time (**Fig 1B–1D**). At each time point, the lung injury scores of each group were calculated, which included alveolar and interstitial inflammation, pulmonary hemorrhage, edema, atelectasis, and formation of hyaline membranes. A significantly higher score was found in LPS groups than the saline group (2h: 3.13±0.64 *vs*. 0.5±0.53, 6h: 6.75±1.39 *vs*. 0.75±0.46, 12h: 15±1.85 *vs*.1.13±0.64; p<0.01) (**Fig 1E**). Consistent with pathological changes, PaO_2_ in LPS groups was significantly lower compared with control groups (2h: 85.14±2.67 *vs*. 91.64±6.50, 6h: 72.20±4.03 *vs*. 87.47±3.87, 12h: 49.11±7.59 *vs*. 85.99±3.72 mmHg; p<0.05). At 12 h, PaO_2_ in the LPS group was less than 60 mmHg (**Fig 1F**). Based on the results of pathology and PaO_2_, our ARDS model was established to mimic the acute phase of human ARDS.

### CXCL9, CXCL10, and CXCL11 levels in LPS-induced ARDS

**Fig 2:**
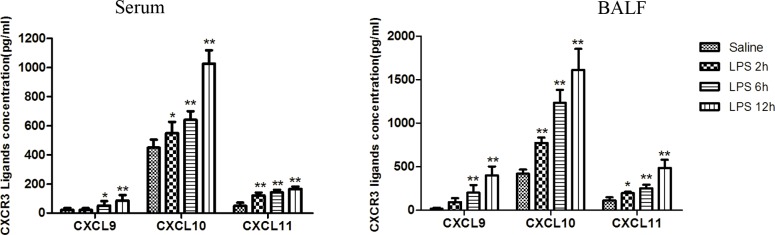
Elevated CXCL9, CXCL10, and CXCL11 expression in LPS-induced ARDS in rats. After the indicated time of LPS or saline injection, blood and BALF were collected. CXCL9, CXCL10, and CXCL11 in both serum and BALF were analyzed using ELISA. Results are shown as mean±SD, n = 6–8 for each group. **p<0.01, *p<0.05 compared with the control. ARDS: acute respiratory distress syndrome; BALF: bronchoalveolar lavage fluid; ELISA: enzyme-linked immunosorbent assay.

To examine CXCR3 ligands (CXCL9, CXCL10, and CXCL11) expression in LPS-induced ARDS, we measured the three chemokines in serum and BALF using ELISA. LPS-induced ARDS in rats led to significant increase in the expression of CXCL9, CXCL10, and CXCL11 in serum and BALF compared with saline control rats (p<0.05) and they further increased with longer exposure to LPS. Compared with saline group, 12 h after LPS administration, serum and BALF levels of CXCL10 (1026.87±91.26 *vs*. 449.93±55.9 pg/ml in serum, 1611.17±243.01 *vs*. 418.93±48.81 pg/ml in BALF, p<0.01), CXCL9 (86.21±37.97 *vs*. 22.6±14.07 pg/ml in serum, 401.13±102.06 *vs*.17.45±11.13 pg/ml in BALF, p<0.01) and CXCL11 (166.26±16.84 *vs*. 50.54±21.64 pg/ml in serum, 485.47±93.34 *vs*. 111.36±38.31 pg/ml in BALF, p<0.01) were significantly increased. Furthermore, the levels of CXCL10 increased much more than CXCL9 and CXCL11 (**Fig 2**). Therefore, compared with CXCL9 and CXCL11, CXCL10 may play a major role in the progression of ARDS induced by LPS.

### Upregulation of CXCL10 and its receptor CXCR3 in LPS-induced ARDS in lung tissue

**Fig 3:**
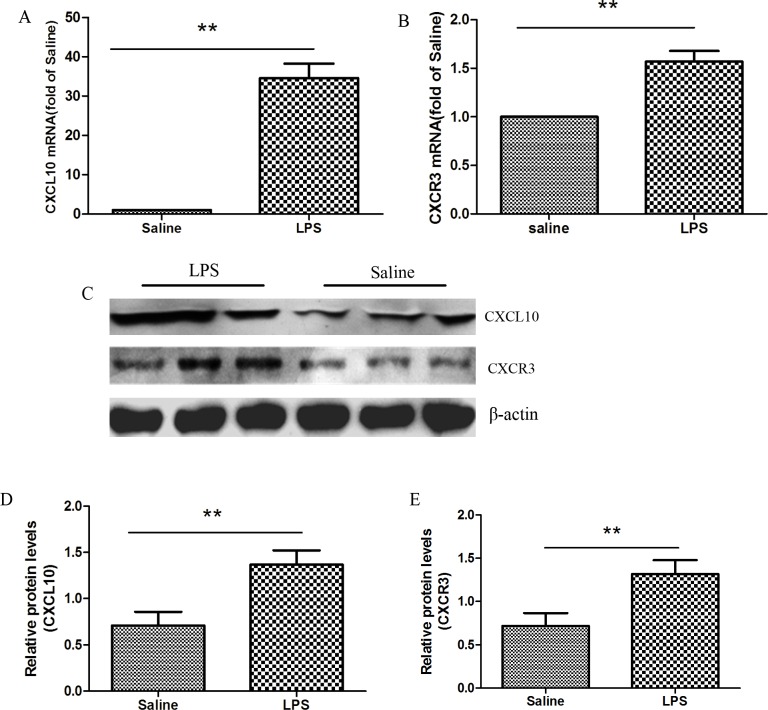
Elevated CXCL10 and CXCR3 expression in the lung tissues in ARDS model. 12 h after LPS or saline was administered, the right lower lungs were obtained. (A, B) qPCR was performed to analyze CXCL10 and CXCR3 mRNA expression in the lung tissues. (C-E) Western blotting was performed to measure CXCL10 and CXCR3 protein expression in lung tissues. Each bar represents the mean±SD of 6–8 rats. **p<0.01 compared with control group. qPCR: quantitative polymerase chain reaction.

CXCL10 is known to exert its function by binding to its receptor CXCR3. We explored CXCL10 and CXCR3 expression in lung tissues using both q-PCR and Western blotting. After 12 h of LPS injection, the relative mRNA expression of CXCL10 and CXCR3 (34.60±9.76 and 1.57±0.32 respectively) were significantly increased compared with the saline control group (p<0.01) (**Fig 3A and 3B**).The protein expression of CXCL10 (1.41±0.11 *vs*. 0.73±0.11, p<0.01) and CXCR3 (1.32±0.16 *vs*. 0.72±0.15, p<0.01) were agreed with mRNA expression levels **(Fig 3C–3E)**.

### Neutralization of CXCL10 decreased pulmonary edema in LPS-induced ARDS

**Fig 4:**
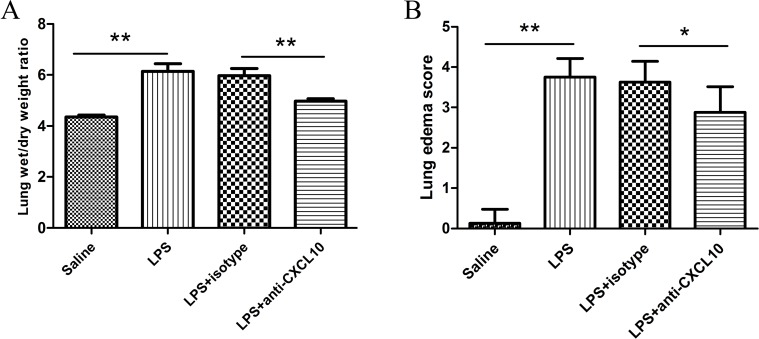
Neutralization of CXCL10 decreased pulmonary edema in LPS induced ARDS. (A) Lung wet/dry weight ratio and (B) lung edema score are presented as the mean±SD (n = 8 in each group). **p<0.01 between the indicated groups.

With increased time, the lung injury worsened and the level of CXCL10 progressively increased. Based on our results, we next examined whether CXCL10 neutralization could ameliorate the severity of ARDS. Lung wet/dry weight ratio (W/D) is an indicator of pulmonary vascular permeability to water. As shown in **Fig 4A**, after LPS administration, the lung W/D ratio increased significantly compared with the saline group (6.15±0.72 *vs*. 4.35±0.24, p<0.01), while administration of anti-CXCL10 antibody significantly decreased the lung W/D ratio compared with the LPS+isotype control group (4.98±0.25 *vs*. 5.97±0.81, p<0.01), and there was no difference between the LPS groups and LPS+isotype control group (p>0.05). Consistent with W/D, the lung edema score in the LPS group increased significantly compared with the saline group (3.75±0.46 *vs*. 0.13±0.35, p<0.01) and a significantly decreased lung edema score was detected after further administration of anti-CXCL10 antibody compared with anti-IgG1 antibody (2.88±0.64 *vs*. 3.63±0.52, p = 0.02) (**Fig 4B)**. These results suggest that neutralization of CXCL10 can improve lung function in ARDS.

### Neutralization of CXCL10 decreased the gene expression of inflammatory mediators in LPS-induced ARDS

**Fig 5:**
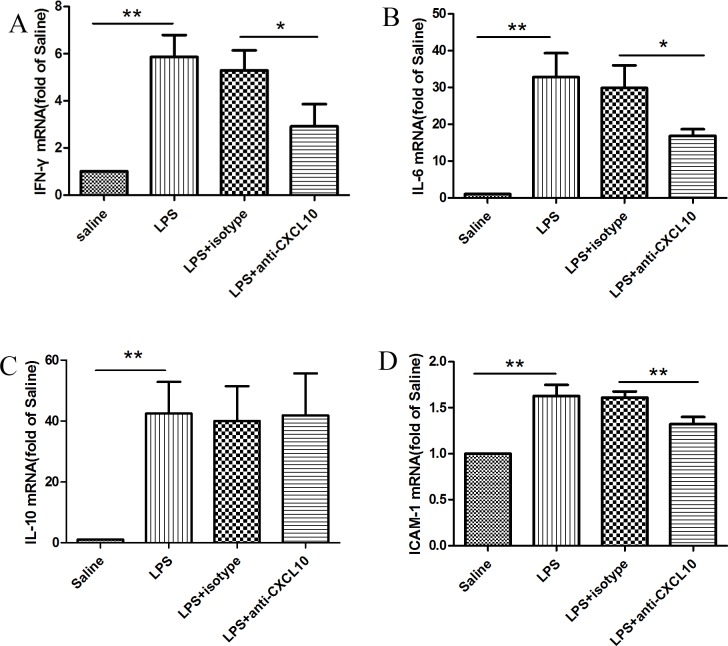
mRNA expression of IFN-γ, IL-6, IL-10, and ICAM-1. The rats were divided into four groups: saline group, LPS group, LPS+isotype control group, and LPS+anti-CXCL10 Ab group. After 12 h interventions, total RNA was extracted from lung tissues and mRNA expression for IFN-γ(A), IL-6(B), IL-10(C), and ICAM-1(D) was quantified using qPCR. The data for mRNA expression were presented as 2^−ΔΔCt^ values. Results are shown as mean±SD (n = 6–8). **p<0.01,*p<0.05 between the indicated groups.

To evaluate whether CXCL10 regulates the gene expression of inflammatory mediators involved in the development of ARDS, the mRNA levels of IFN-γ, IL-6, IL-10 and ICAM-1 were measured. LPS induction significantly upregulated mRNA expression of IFN-γ (5.86±2.47), IL-6 (32.82±15.86), IL-10 (42.46±10.44), and ICAM-1(1.63±0.12) (p<0.01). Furthermore, the rats pretreated with monoclonal anti-CXCL10 antibody showed significantly lower levels of IFN-γ, IL-6 and ICAM-1compared with the LPS group or LPS+isotype control group (IFN-γ: 2.92±2.49 *vs*. 5.29±2.27, IL-6:16.87±4.64 *vs*. 29.87±16.19, ICAM-1:1.32±0.08 *vs*. 1.61±0.06, p<0.05). In contrast, CXCL10 neutralization did not suppress IL-10 mRNA expression **(Fig 5**).

### Neutralization of CXCL10 decreased inflammatory cells recruitment in LPS-induced ARDS

**Fig 6:**
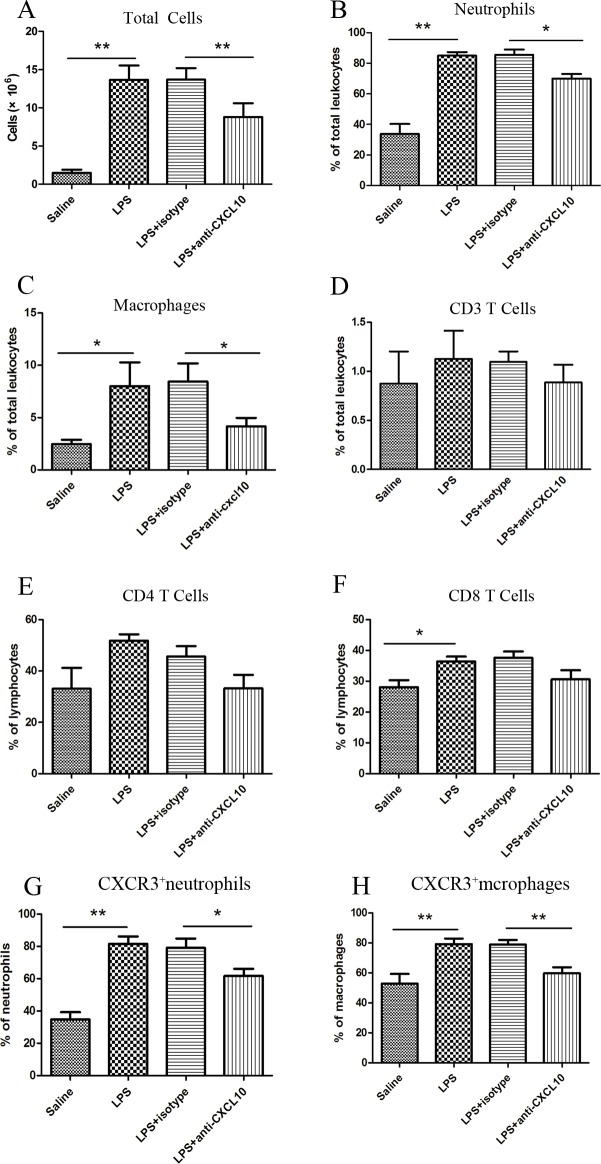
Cell counts in BALF in the four divided groups by flow cytometry. After 12 h interventions, the left lungs were lavaged. BALF was analyzed by measuring total cells, neutrophils, macrophages, lymphocytes, CXCR3+neutrophils and CXCR3+ macrophages. Results are shown as mean±SD (n = 6–8). **p<0.01,*p< 0.05 between the indicated groups.

**Fig 7:**
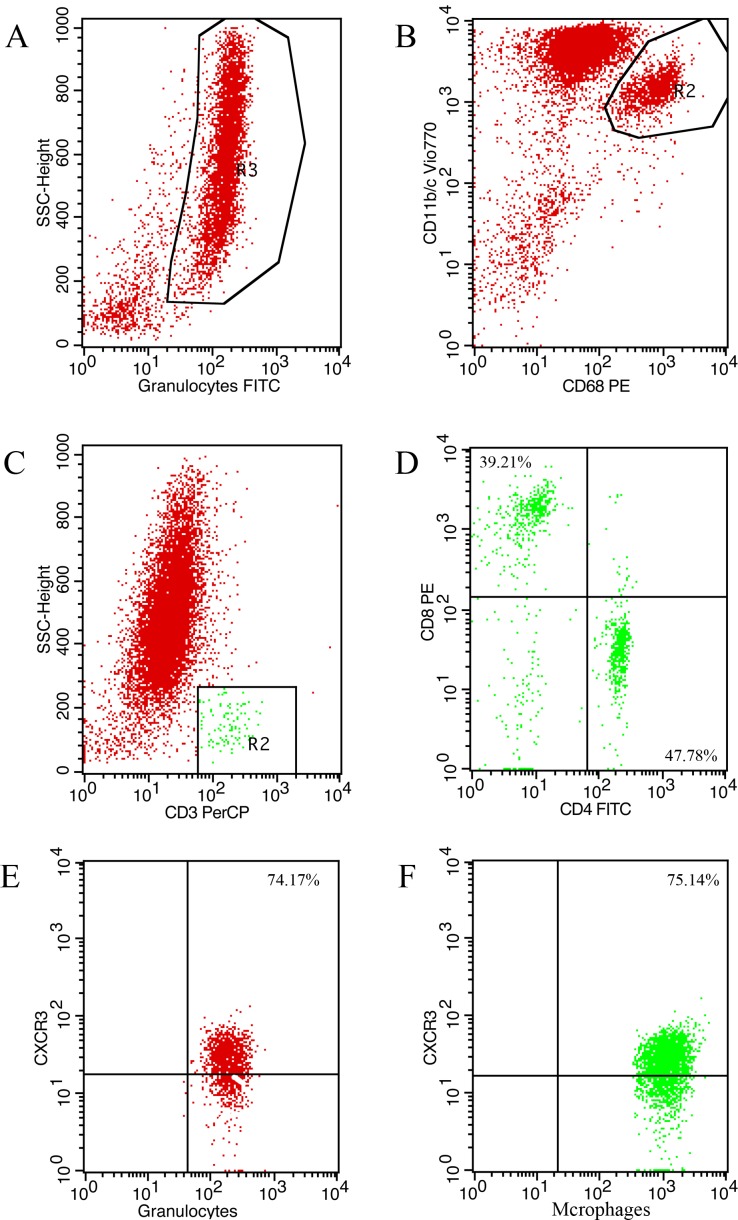
Representative flow cytometric plots. (A) neutrophils, (B) macrophages, (C) CD3, (D) CD4/CD8, (E) CXCR3+ neutrophils, and (F) CXCR3+ macrophages.

We utilized flow cytometry to investigate total cells, neutrophils, macrophages and T lymphocytes changes in BALF. T lymphocytes were separated by CD3 and then further delineated by CD3+CD4+ and CD3+CD8+. After LPS was given, the total cell numbers in BALF were significantly increased compared with the saline control group (13.66±1.87×10^6^ *vs*. 1.49±0.41×10^6^, p<0.01). The absolute number and the percent of neutrophils (11.57±1.41×10^6^ and 85.05±6.64% *vs*. 0.54±0.39×10^6^ and 33.81±18.24%, p<0.01), macrophages (10.79±7.56×10^5^ and 8.02±5.54% *vs*. 0.37±0.19×10^5^and 2.45±1.05%, p<0.05) and CD3+CD8+ lymphocytes (5.0±3.77×10^4^and 36.46±4.17% *vs*. 0.44±0.48×10^4^and 28.12±5.53%, p<0.05) also significantly increased, while there was no difference in CD3+T lymphocytes and CD3+CD4+T lymphocytes between the two groups (p>0.05). After CXCL10 was blocked, total cells (8.80±1.81×10^6^ *vs*. 13.71±1.48×10^6^, p<0.01), neutrophils (6.13±1.34×10^6^ and 70±8.11% *vs*. 11.7±1.37×10^6^ and 85.66±8.73%, p<0.01) and macrophages (3.57±1.24×10^5^ and 4.16±1.98% *vs*. 11.89±6.25×10^5^ and 8.45±4.25%, p<0.05) but not CD3+CD8+ T cells decreased compared with the isotype control group (**Fig 6A–6F**). As the majority of leukocytes in BALF after LPS induction were neutrophils and macrophages, we next investigated the proportion of CXCR3 positivity in neutrophils and macrophages. The percent of CXCR3+neutrophils (81.5±11.57% *vs*. 34.79±10.75%, p<0.01) and CXCR3+macrophages (79.26±9.12% *vs*. 52.95 ± 15.59%, p<0.01) were significantly elevated in the LPS group compared with the saline group (p<0.01), moreover, treatment with anti-CXCL10 reduced the proportion of CXCR3+ cells among neutrophils (61.75±10.97% *vs*. 79.12±14.28%, p<0.05) and macrophages (59.76±9.84% *vs*. 79.06±7.21%, p< 0.01) compared with the isotype control group (**Fig 6G and 6H**). The representative flow cytometric plots are shown in **Fig 7**.

## Discussion

In the present study, we found that neutralization of CXCL10 could ameliorate the severity of ARDS induced by LPS. The molecular mechanisms associated with the effect of CXCL10 were that CXCL10 could promote the development of LPS-induced ARDS by increasing pro-inflammatory cytokines (IFN-γ, IL-6) and intercellular adhesion molecule (ICAM-1) expression in lung tissues. Moreover, CXCL10 could induce inflammatory cells migration to the lung and exacerbate the pathology of ARDS. After pretreatment with anti-CXCL10 Ab, pulmonary edema and inflammation were significantly alleviated. The CXCL10-CXCR3 axis also contributed to the progression of ARDS. In addition to the elevated CXCL10 and CXCR3 mRNA and protein levels in ARDS, CXCL10 neutralization reduced the total cells as well as the percentage of neutrophils and macrophages, accompanied with reduced CXCR3+ neutrophils and CXCR3+ macrophages infiltration. Therefore, our study supported that CXCL10 plays a key role in the development of ARDS after LPS induction.

CXCL10, also known as IFN-γ inducible protein 10 (IP-10), is a pro-inflammatory factor that can be induced by IFN-γ as well as some other factors including LPS and TNF-α [25, 26]. CXCL10 could amplify the effects of other cytokines [27]. Numerous studies have shown that pro-inflammatory and anti-inflammatory cytokines are critical mediators associated with the development of ARDS [6, 28, 29]. These inflammatory mediators may be secreted from cells recruited into the air spaces in response to the inflammatory cascade. We evaluated whether CXCL10 could affect IL-6, IL-10, IFN-γ, and ICAM-1 involved in ARDS. Our results indicated that CXCL10 was correlated with the expression of IL-6, IFN-γ, and ICAM-1. Although IL-6 activates both pro-inflammatory and anti-inflammatory mechanisms, it primarily possesses pro-inflammatory actions. The pro-inflammatory cytokine IL-6 is one of the key biomarkers in both ARDS patients and animal models that can predict the morbidity and mortality of ARDS patients [30, 31]. IFN-γ, a Th1 associated pro-inflammatory cytokine, is known to be essential in airway inflammation and inducing the influx of neutrophils [32, 33]. As a major inducer of CXCL10, excessive production of IFN-γ and CXCL10 contributed to injury progression in ARDS [34]. In addition to the pro- inflammatory cytokines, intercellular adhesion molecule 1 (ICAM-1) also has an important effect on ARDS by playing a role in trafficking of leukocytes to the site of inflammation [35, 36]. During ARDS, ICAM-1 was upregulated on the lung epithelium and higher levels of ICAM-1 were suggestive of an acute inflammatory process in patients with ARDS, which became more serious with increased mortality [37]. Our data suggest that regulation of pro-inflammatory cytokines and intercellular adhesion molecule by CXCL10 plays a role in the inflammatory response in LPS-induced ARDS.

The recruitment of leukocytes to inflammatory lung tissues is essential for the development of ARDS. After being released in the blood, leukocytes migrate to the site of inflammation, requiring adhesion and transmigration through blood-vessel walls. Large amounts of chemokines and cytokines released by endothelial, epithelial, and inflammatory cells are involved in this process [38]. Because neutrophil-mediated lung injury plays a pivotal role in ARDS, chemokines that may contribute to neutrophil chemotaxis were further studied. Some chemokines including CXCL8, C-C motif chemokine ligand 2 (CCL2), and CCL7 are known to enhance neutrophil activation and migration [10, 39]. Besides chemokines, peripheral blood monocytes, along with alveolar macrophages may influence neutrophil migration and their depletion prior to LPS exposure can diminish neutrophil trafficking. Therefore, macrophages may direct neutrophil-mediated lung injury in the LPS-induced ARDS model [40, 41]. In our model, neutrophil and macrophages in BALF were significantly increased after administration of LPS, while inhibition of CXCL10 decreased the recruitment of neutrophils and macrophages into the alveoli. Therefore, our results indicated that CXCL10 was associated with the recruitment of neutrophils and macrophages in ARDS.

CXCL10 exerts its functions by binding to its receptor CXCR3, a G-protein-coupled receptor, which has been demonstrated to be expressed on multiple cell types including lymphocytes and monocytes [42]. It was generally believed that CXCR3 was not expressed on neutrophils, while recent studies showed that CXCR3 was present on neutrophils in acute lung injury and chronic lung disease. Furthermore, the CXCL10-CXCR3 axis could activate oxidative bursts and chemotaxis in neutrophils in a possible autocrine loop [12, 43]. Remarkably, local tissue secretion of CXCL10 represents the driving force for the recruitment of CXCR3 positive inflammatory cells. In our study, elevated mRNA and protein levels of CXCL10 and CXCR3 in lung tissues were measured using quantitative PCR and Western blotting. CXCR3 expression in BALF macrophages and neutrophils was increased in LPS-induced ARDS and CXCL10 neutralization reduced CXCR3 positive cells infiltration. Taken together, our results indicated that the CXCL10-CXCR3 axis contributed to the development of ARDS. Moreover, reducing the migration of CXCR3 positive cells into inflammatory sites played a role in CXCL10 neutralization in LPS-induced ARDS. Further studies on the effect of CXCL10 in LPS-induced ARDS at the cellular level are required.

## Conclusion

Our results demonstrated that the chemokine CXCL10 promoted the development of ARDS by binding to its receptor CXCR3. Moreover, CXCL10 neutralization ameliorated LPS-induced ARDS by suppressing the recruitment of inflammatory cells into the lungs and reducing the induction of inflammatory mediators involved in the pathology of ARDS. Thus, CXCL10 could be a potential therapeutic target for the treatment of LPS-induced ARDS.

## Supporting Information

S1 Information Minimal data set for Figs 1–6. (XLSX)
